# Variability of test parameters from mice of different age groups in published data sets

**DOI:** 10.1371/journal.pone.0329357

**Published:** 2025-08-07

**Authors:** Bernhard Aigner

**Affiliations:** Chair for Molecular Animal Breeding and Biotechnology, LMU Munich, Munich, Germany; Ehime University Graduate School of Medicine, JAPAN

## Abstract

The use of mice as animal models in biomedical research allows the standardization of genetic background, housing conditions as well as experimental protocols, which all affect phenotypic variability. In this study, the phenotypic variability of test parameters was analyzed in genetically identical mice of different age groups, i.e., early adults versus late adults. Therefore, published data sets of genetically identical mice of different age groups collected from the same investigator/ project were retrospectively analyzed. Morphological parameters, blood parameters and behavioral tests were analyzed which are predominantly used in biomedical research. The JaxKOMP project examined C57BL/6NJ mice with an age of 7–20 weeks and 66–81 weeks. Further substrains of C57BL/6N mice with an age of 8–16 weeks and 49–63 weeks were examined as wild-type controls from various investigators of the International Mouse Phenotyping Consortium (IMPC). Additional data sets of young and old groups of genetically identical mice were derived from the Mouse Phenome Database (MPD) and the RIKEN BioResource Research Center (RBRC). The phenotypic variability of the chosen traits and parameters was measured by calculating the coefficient of variation (CV = standard deviation/ mean) of the animals with the same sex of a given mouse strain. Subsequently, the CVs of the young and the old mouse group were compared. The comparison of the phenotypic variability of the late adults versus early adults revealed the appearance of unpredictable interactions between genotype, environment and experiment. Overall, a higher phenotypic variability of the late adults appeared almost consistently for body weight including lean mass and fat mass for females as well as for hematology and immunology parameters, particularly for females. Clinical chemistry often appeared inconspicuous. No noticeable differences were detected for the traits echocardiography and electrocardiogram, whereas late adults also often showed a higher phenotypic variability for behavioral tests.

## Introduction

Phenotypic variability of genetically identical individuals is caused by known or unrecognized unstandardized abiotic and biotic environmental conditions as well as by stochastic cellular noise leading to phenotypic variability in individual mice which gives rise to an intrinsic variance that cannot be reduced [1]. Inbred mouse strains offer the opportunity to address the question if phenotypic variability increases in older mice as a consequence of the prolonged impact of these factors. The phenotypic variability within the experimental unit determines the choice of the group size which is necessary for achieving valid and reproducible results. Literature search for publications dealing with age-related phenotypic variability as primary study goal and by using sufficient animal numbers resulted in the absence of such studies up-to-date.

In biomedical research, usually inbred mice are used as young adults with an age of 6–12 weeks [2]. Morphological parameters, blood parameters and behavioral tests of young adult mice as well as of older mouse groups were examined to detect age-specific differences for chosen phenotypic parameters. The data were collected from the same investigator/ project by using standardized experimental protocols, ideally in the same mouse cohort after further carrying out husbandry in the same standardized environment until the recurrent analysis of the chosen parameters. Such data were also delivered to public databases to serve as reference values for wild-type mouse strains at a higher age.

In this study, the phenotypic variability of test parameters in genetically identical mice of different age groups (early adults versus late adults) was analyzed in data sets of laboratory mouse strains which have been submitted to the Mouse Phenome Database (https://phenome.jax.org/centers/JaxKOMP; https://phenome.jax.org), the International Mouse Phenotyping Consortium (IMPC) (https://www.mousephenotype.org/data/late-adult-data), and the RIKEN BioResource Research Center (RBRC) (https://ja.brc.riken.jp/lab/jmc/mouse_clinic/Aged_JMC/group_Condition-Aged_mice/index.html). Parameters with sufficient data submitted to the databases were selected for this study which may indicate the frequency of the use of the respective parameters in biomedical research with mice.

## Materials and methods

Public mouse databases were searched for data sets of genetically identical groups of different age periods which have been collected from the same investigator/ project by using standardized experimental protocols, ideally in the same animal cohort. Morphological parameters, blood parameters and behavioral tests were analyzed. In this study, body weight as a test parameter turned out to show a different variability in different age groups (see “Results” section), therefore, further test parameters were compared between the age groups as absolute values without relation to body weight or body composition parameters.

The JaxKOMP project (https://phenome.jax.org/centers/JaxKOMP) analyzed C57BL/6NJ mice with an age of 7–20 weeks (“early adult”) and 66–81 weeks (“late adult”). A great number of test parameters were examined in a great number of mice, and all results of the individual mice have been submitted online. Therefore, in the current study the 95% data range (by excluding 2.5% each of the highest and lowest values) was chosen for each parameter as physiological range to exclude technical outliers. Beside phenotypic traits analyzed as single parameter, the other traits included following parameters (VT, vertebrate trait ontologies): bone (n = 3; bone mineral content (BMC), bone mineral density (BMD), bone area); clinical chemistry (n = 13; albumin, VT:0000199; bilirubin, VT:0001569; cholesterol, VT:0000180; creatinine, VT:0005328; glucose, VT:0000188; total protein, VT:0005567; triglycerides, VT:0002644; urea, VT:0005265; calcium, VT:0001562; phosphorus, VT:0001565; alanine aminotransferase (ALT, EC 2.6.1.2), VT:0001573; aspartate aminotransferase (AST, EC 2.6.1.1), VT:0000203; alkaline phosphatase (AP, EC 3.1.3.1)); glucose tolerance test (n = 5; plasma glucose 0 min, 15 min, 30 min, 60 min, 120 min); hematology (n = 7; hemoglobin (MHEM), VT:0001588; mean corpuscular volume (MCV), no VT applied; red blood cell count (RBC), VT:0001586; white blood cell count (WBC), VT:0000217; platelets (PLT), VT:0003179; RBC corpuscular distribution width (RDW), no VT applied; mean platelet volume (MPV), no VT applied. The parameters hematocrit, mean corpuscular hemoglobin (MCH), and mean corpuscular hemoglobin concentration (MCHC) were not included in the study as they are subsequently calculated by using parameters which are directly measured.); electrocardiogram (n = 12; heart rate, heart rate variability, CV, RR interval, PQ interval, PR interval, QRS complex duration, QT interval, ST segment, QTc dispersion, mean SR amplitude, mean R amplitude); grip strength (n = 2; forelimb grip strength mean, fore- and hindlimb grip strength mean); light-dark box (n = 8; reaction time, side changes, left side time spent, left side mobile time spent, right time side spent, right side mobile time spent, percent time in light, percent time in dark); and open field (n = 19; time spent mobile, distance traveled total, periphery distance traveled, center distance traveled, number of rears first five minutes, corners permanence time, percent time center, whole arena resting time, whole arena average speed, periphery resting time, periphery permanence time, periphery average speed, center resting time, center permanence time, center average speed, number of center entries, vertical time, number of rears total, percent time corners).

Further C57BL/6N substrains (C57BL/6N, C57BL/6NCrl, C57BL/6NJcl, C57BL/6NTac) with an age of 8–16 weeks (“early adult”) and 49–63 weeks (“late adult”) were examined as wild-type controls from various investigators (Baylor College of Medicine (BCM), Helmholtz-Zentrum Muenchen (HMGU GMC; two projects with two substrains), Institut Clinique de la Souris, France (ICS), Korea Mouse Phenotyping Center (KMPC), Mary Lyon Centre at MRC Harwell, RIKEN BioResource Research Center (RBRC), The Centre for Phenogenomics Canada (TCP), UC Davis) of the International Mouse Phenotyping Consortium (IMPC) following standardized phenotyping pipelines, as described in IMPReSS (https://www.mousephenotype.org/data/late-adult-data). Only the data of the wild-type controls but no data of genetically modified mouse lines were used. Data of the Jackson Laboratory (JAX) in this database were not used as data of the JaxKOMP project was already analyzed in this study (see above). Number of mice tested, mean, and standard deviation (SD) are published online for the parameters tested. Data sets were included with at least 50 mice of a given sex examined as “late adult” group for a test parameter. The test parameters, which were selected from the database and included in Tables 2 and S1, are listed in S2 Table.

**Table 1:** Variability of test parameters from C57BL/6NJ mice of two different age groups of the project JaxKOMP-EAP/ JaxKOMP-LAP (95% range of the data sets).

		f							m						
trait	n param.	late adult (66–81 weeks)		early adult (7–20 weeks)		CV late/ (CV late + CV early)			late adult (67–81 weeks)		early adult (7–20 weeks)		CV late/ (CV late + CV early)		
		n	CV (range)	n	CV (range)	mean	range	r > 0.5 (n)	n	CV (range)	n	CV (range)	mean	range	r > 0.5 (n)
body weight	1	508	0.16	675	0.07	0.71			516	0.15	666	0.08	0.65		
DXA body weight	1	324	0.16	2468	0.08	0.66			326	0.16	2403	0.10	0.61		
DXA lean mass	1	324	0.09	2468	0.07	0.55			326	0.10	2403	0.10	0.51		
DXA fat mass	1	324	0.33	2468	0.23	0.59			326	0.35	2403	0.30	0.54		
body length	1	324	0.03	2468	0.03	0.52			326	0.03	2402	0.03	0.49		
heart weight	1	329	0.10	2244	0.10	0.49			326	0.10	2165	0.13	0.43		
bone	3	324	0.05-0.10	2468	0.05-0.10	0.50	0.46-0.55	1 of 3	326	0.05-0.09	2403	0.06-0.12	0.46	0.42-0.53	1 of 3
clinical chemistry	13	212+	0.03-0.38	898+	0.03-0.65	0.49	0.28-0.73	6 of 13	207+	0.03-0.54	879+	0.02-0.52	0.50	0.42-0.64	7 of 13
glucose tolerance	5	310	0.16-0.25	2391	0.15-0.19	0.53	0.45-0.61	3 of 5	311	0.18-0.30	2345+	0.17-0.19	0.55	0.50-0.61	5 of 5 *
hematology (total)	7	210	0.03-0.39	1970+	0.02-0.32	0.59	0.47-0.70	6 of 7	204	0.03-0.35	1910+	0.02-0.28	0.56	0.45-0.64	6 of 7
- hematology	5	210	0.03-0.15	1973+	0.02-0.13	0.57	0.47-0.67	4 of 5	204	0.03-0.15	1910+	0.02-0.15	0.55	0.45-0.60	4 of 5
- WBC, platelets	2	210	0.21-0.39	1970+	0.09-0.32	0.63	0.55-0.70	2 of 2	204	0.18-0.35	1912+	0.10-0.28	0.60	0.55-0.64	2 of 2
electrocardiogram	12	142	0.02-0.99	1865	0.04-0.74	0.50	0.32-0.60	7 of 12	139+	0.03-1.07	1817	0.04-0.86	0.51	0.40-0.60	7 of 12
grip strength	2	359	0.10-0.12	2500	0.12-0.14	0.46	0.46-0.46	0 of 2	382	0.11-0.13	2464	0.12-0.15	0.48	0.48-0.48	0 of 2
hole board	1	318	0.36	1790	0.29	0.55			304	0.43	1762	0.34	0.56		
light-dark box	8	338	0.23-1.08	2434	0.13-0.95	0.60	0.53-0.68	8 of 8 **	356	0.21-0.85	2392	0.17-0.86	0.60	0.50-0.67	7 of 8 *
open field	19	129+	0.18-0.70	433	0.12-0.65	0.55	0.46-0.74	15 of 19 *	131+	0.20-0.89	429	0.14-0.84	0.59	0.51-0.74	19 of 19 ***

Data of the JaxKOMP project (https://phenome.jax.org/centers/JaxKOMP) were used for the analysis. The 95% data range (by excluding 2.5% each of the highest and lowest values) was chosen for each test parameter separately as physiological range to exclude technical outliers.

For each test parameter, the CV of the early adult group and the CV of the late adult group, and subsequently the ratio r = CV_late adult_/ (CV_late adult_ + CV_early adult_) was calculated separately. For traits with more than one test parameter measured (bone, clinical chemistry, glucose tolerance, hematology, electrocardiogram, grip strength, light-dark box, open field), the mean of the r’s of all test parameters of a given trait was calculated (see “mean”). Two additional calculations are shown: the range of the r’s of all test parameters of a given trait (see “range”). And “r > 0.5 (n)”: The number of the test parameters where the ratio r = CV_late adult_/ (CV_late adult_ + CV_early adult_) is > 0.5. In the case that the late adults show the identical phenotypic variability as the early adults, 50% of the parameters of a given trait are expected to deliver r values > 0.5 (and the other 50% of the parameters are expected to deliver r values < 0.5). The detected counts of r values > 0.5 and < 0.5 were statistically analyzed by using the chi-squared test (*, p < 0.05; **, p < 0.01; ***, p < 0.001).

The trait “hematology (total)” included seven test parameters (see Materials and Methods section). As the two parameters “WBC” and “platelets” usually showed much higher CVs within a given mouse group vs. the other five remaining hematology parameters, separate r values are in addition listed for the five parameters showing a low CV (“hematology”), as well as for WBC and platelets alone (“WBC, platelets”).

f, female; m, male; n, number of mice examined in the age group (n + : In the case that different group sizes were analyzed for the various test parameters of a given trait, the group size with the lowest animal number is shown in the table); CV, coefficient of variation = standard deviation/ mean; DXA, dual-energy X-ray absorptiometry; WBC, white blood cell count.

**Table 2:** Variability of test parameters from C57BL/6N mice (n ≥ 50 per sex for the late adult group) of two different age groups of the database IMPC LATE ADULT.

			f					m				
trait	n projects	n param.	n late adult (49–63 weeks)	n early adult (8–16 weeks)	CV late/ (CV late + CV early)			n late adult (49–63 weeks)	n early adult (8–16 weeks)	CV late/ (CV late + CV early)		
					mean	median	range (projects)			mean	median	range (projects)
body weight	9	1	63-4390	395-19266	0.57		0.48-0.65	62-3627	417-20602	0.49		0.38-0.60
DXA lean mass	6-7	1	55-376	314-1890	0.54		0.47-0.61	62-346	316-1884	0.49		0.43-0.54
DXA fat mass	6-7	1	55-374	314-1890	0.52		0.43-0.62	62-346	316-1884	0.50		0.39-0.64
body length	4-5	1	63-284	395-834	0.48		0.38-0.65	62-284	388-780	0.44		0.24-0.58
tibia length	2	1	86-409	667-1113	0.49		0.46-0.53	80-410	660-1082	0.51		0.49-0.53
heart weight	9	1	63-549	163-2257	0.48		0.40-0.51	55-450	163-2222	0.45		0.31-0.55
bone	6-7	3	55-377	314-1890	0.56	0.55	0.47-0.64	62-290	316-1884	0.56	0.56	0.51-0.59
clinical chemistry	5	12-20	102-555	223-2214	0.51	0.51	0.49-0.53	93-485	217-2198	0.53	0.52	0.51-0.57
IPGTT	7	2 3	63-600	556-2667	0.55	0.55	0.52-0.62	62-512	543-2662	0.56	0.56	0.50-0.62
insulin	1	1	72	344	0.53			77	363	0.57		
hematology (total)	5-6	8-10	58-290	276-1229	0.54	0.53	0.50-0.58	80-242	278-1244	0.50	0.50	0.45-0.54
- hematology	5-6	6-8	58-289	276-1229	0.52	0.51	0.49-0.55	80-241	284-1244	0.49	0.49	0.43-0.53
- WBC, platelets	5-6	1-2	58-290	281-1228	0.62		0.52-0.67	80-242	278-1240	0.54		0.52-0.58
immunology	2	62-68	62-63	135-171	0.57	0.56	0.51-0.62	58-62	135-167	0.53	0.54	0.51-0.56
spleen weight	3	1	79-549	431-2252	0.68		0.65-0.73	68-449	402-2219	0.50		0.46-0.59
echocardiography	3	9-14	59-348	774-1296	0.52	0.52	0.49-0.55	63-297	183-995	0.50	0.50	0.47-0.53
electrocardiogram	6	6-8	57-507	300-1546	0.49	0.50	0.46-0.52	53-398	264-1563	0.50	0.51	0.44-0.57
grip strength	7	2	60-551	754-2748	0.47		0.43-0.51	50-449	745-2763	0.49		0.45-0.51
fear conditioning	2	19-31	140-358	288-290	0.45	0.46	0.45-0.45	139-311	258-292	0.44	0.43	0.43-0.45
light-dark box	1	3	115	783	0.45	0.49		113	722	0.50	0.51	
open field	4	6-16	106-476	632-2388	0.53	0.52	0.52-0.54	108-406	627-2379	0.53	0.53	0.52-0.56
rotarod	1	1	106	767	0.55			107	837	0.53		
Y-maze	1	9	202	105	0.57	0.56		194	104	0.59	0.60	

Data of the database IMPC LATE ADULT (https://www.mousephenotype.org/data/late-adult-data) were used for the analysis. Data of up to nine projects using wild-type animals of C57BL/6N substrains with an age of 8–16 weeks (“early adult”) and 49–63 weeks (“late adult”) were used. Data sets were included with at least 50 mice of a given sex examined as “late adult” group for a test parameter. The test parameters chosen from the database are listed in S2 Table.

If project data sets were included only for one sex, this is indicated by different project numbers shown for the trait concerned (see “n projects”). In the case that different group sizes were analyzed for the various test parameters of a given trait in a project, the group size with the lowest animal number was used for the determination of the range of the animal numbers used across all projects (see “n late adult (49-63 weeks)” and “n early adult (8-16 weeks)”).

Within each project, for each test parameter, the CV of the early adult group and the CV of the late adult group, and subsequently the ratio r = CV_late adult_/ (CV_late adult_ + CV_early adult_) was calculated separately. For traits with more than one test parameter measured (bone, clinical chemistry, IPGTT, hematology, immunology, echocardiography, electrocardiogram, grip strength, fear conditioning, light-dark box, open field, Y-maze), the mean and the median of the r’s of all test parameters within a given trait of a project was calculated. Subsequently, the mean was calculated from the project means (see “mean”). Additionally, for traits with more than one test parameter also the mean of the project medians was calculated (see “median”), and the range of the project means is shown (see “range (projects)”).

The trait “hematology (total)” included 8–10 test parameters (see S2 Table). As the two parameters “WBC” and “platelets” usually showed much higher CVs within a given mouse group vs. the other remaining hematology parameters, separate r values are in addition listed for the remaining parameters showing a low CV (“hematology”), as well as for WBC and platelets alone (“WBC, platelets”).

f, female; m, male; n, number of mice examined in the age group; CV, coefficient of variation = standard deviation/ mean; DXA, dual-energy X-ray absorptiometry; IPGTT, intraperitoneal glucose tolerance test; WBC, white blood cell count.

Additional data sets of young and old groups of genetically identical mice were derived from the Mouse Phenome Database (MPD, https://phenome.jax.org). For each phenotypic trait/ parameter, strain data sets were chosen for the study by using the following selection criteria: inbred strains (including those derived from the Collaborative Cross (CC)), F1 hybrids; no lines with newly generated alleles; no treatment; group size: n ≥ 5 of a given sex for a chosen strain in both age groups; data of at least five strains available. Data for the following comparisons of different age groups were found: “6 months old” vs. “18-20 months old”; and “6 months old” vs. “24 months old”. More than one test parameter was analyzed for the phenotypic traits “bone” (n = 3 parameters), “clinical chemistry” (n = 11), “hematology” (n = 6), “immunology” (n = 8–10), “electrocardiogram”(n = 6), and “gait analysis”(n = 16; including data of only three strains). The information about the selected parameters is accessible online in the Mouse Phenome Database (https://phenome.jax.org) under the name of the phenotypic trait and the project name (usually the name of the investigator) given in Table 3.

**Table 3:** Variability of test parameters from mouse strains of two different age groups in data sets (n ≥ 5 per sex) of the Mouse Phenome Database.

trait	n param.	late adult project	early adult project	f CV late/ (CV late + CV early)					m CV late/ (CV late + CV early)				
				mean	median	range (strains)	n strains	r > 0.5 (n)	mean	median	range (strains)	n strains	r > 0.5 (n)
DXA body weight	1	Ackert1 20 mo	Ackert1 6 mo	0.57	0.60	0.31-0.81	23	16 of 23	0.52	0.55	0.15-0.76	19	13 of 19
DXA lean mass	1	Ackert1 20 mo	Ackert1 6 mo	0.59	0.60	0.31-0.79	23	18 of 23 **	0.52	0.56	0.20-0.72	19	11 of 19
DXA fat mass	1	Ackert1 20 mo	Ackert1 6 mo	0.63	0.64	0.44-0.77	23	20 of 23 ***	0.56	0.59	0.33-0.91	19	12 of 19
body length	1	Ackert1 20 mo	Ackert1 6 mo	0.61	0.62	0.40-0.83	23	19 of 23 **	0.55	0.58	0.16-0.90	19	13 of 19
DXA total area	1	Ackert1 20 mo	Ackert1 6 mo	0.54	0.58	0.30-0.70	23	13 of 23	0.47	0.52	0.13-0.71	19	10 of 19
bone	3	Ackert1 20 mo	Ackert1 6 mo	0.56	0.57	p: 0.53–0.60	23		0.50	0.53	p: 0.47–0.56	19	
- BMC				0.56	0.55	0.28-0.77	23	17 of 23 *	0.47	0.47	0.30-0.74	19	7 of 19
- BMD				0.60	0.62	0.35-0.80	23	18 of 23 **	0.56	0.59	0.28-0.74	19	15 of 19 *
- bone area				0.53	0.54	0.35-0.71	23	13 of 23	0.48	0.52	0.24-0.71	19	11 of 19
clinical chemistry	11	Yuan3 18 mo	Yuan3 6 mo	0.57	0.58	p: 0.50–0.63	17-25		0.54	0.54	p: 0.47–0.60	13-20	
- calcium				0.57	0.56	0.39-0.85	22	15 of 21 *	0.49	0.49	0.15-0.84	13	6 of 13
- chloride				0.55	0.55	0.26-0.82	25	15 of 25	0.54	0.56	0.32-0.80	17	12 of 17
- phosphorus				0.50	0.49	0.22-0.73	25	11 of 25	0.53	0.54	0.27-0.74	16	11 of 16
- potassium				0.50	0.51	0.28-0.70	25	13 of 25	0.47	0.48	0.21-0.67	17	7 of 17
- sodium				0.54	0.53	0.29-0.85	25	15 of 24	0.51	0.52	0.18-0.69	17	10 of 17
- total protein				0.63	0.67	0.29-0.83	22	19 of 22 ***	0.55	0.57	0.30-0.83	20	13 of 20
- HDL cholesterol				0.62	0.66	0.34-0.84	23	17 of 23 *	0.58	0.58	0.31-0.75	20	13 of 20
- urea				0.59	0.60	0.35-0.89	17	12 of 17	0.55	0.57	0.32-0.77	15	11 of 15
- ALP				0.63	0.60	0.32-0.89	25	20 of 25 **	0.60	0.56	0.40-0.85	17	10 of 17
- ALT				0.55	0.62	0.14-0.82	18	11 of 18	0.58	0.56	0.32-0.83	14	11 of 14 *
- lipase				0.56	0.58	0.20-0.76	23	16 of 22 *	0.53	0.54	0.18-0.87	20	12 of 20
hematology	6	Peters4 18 mo	Peters4 6 mo	0.61	0.62	p: 0.58–0.64	25-27		0.57	0.57	p: 0.51–0.64	26-27	
- CHr				0.64	0.64	0.34-0.83	25	23 of 25 ***	0.64	0.67	0.19-0.91	26	22 of 26 ***
- hemoglobin				0.58	0.59	0.28-0.94	27	21 of 27 **	0.53	0.57	0.23-0.83	27	17 of 27
- MCV				0.63	0.61	0.45-0.84	27	25 of 27 ***	0.54	0.54	0.28-0.88	27	16 of 27
- RBC				0.61	0.64	0.29-0.89	27	20 of 27 *	0.60	0.61	0.35-0.86	27	19 of 27 *
- platelets				0.58	0.59	0.20-0.83	27	18 of 27	0.51	0.48	0.19-0.87	27	12 of 27
- WBC				0.63	0.67	0.43-0.83	27	22 of 27 **	0.59	0.57	0.36-0.80	27	22 of 27 **
WBC	1	Petkova1 18 mo	Petkova1 6 mo	0.56	0.54	0.22-0.87	27	17 of 27	0.54	0.55	0.35-0.74	23	15 of 23
immunology	10	Peters4 18 mo	Peters4 6 mo	0.58	0.58	p: 0.54–0.71	27		0.55	0.55	p: 0.44–0.65	26-27	
immunology	8	Petkova1 18 mo	Petkova1 6 mo	0.62	0.63	p: 0.56–0.72	27		0.57	0.57	p: 0.54–0.67	23	
electrocardiogram	6	Xing1 20 mo	Xing1 6 mo	0.51	0.49	p: 0.49–0.54	24		0.52	0.51	p: 0.49–0.56	21	
grip strength	1	Seburn2 18 mo	Seburn2 6 mo	0.51	0.52	0.22-0.70	27	17 of 27	0.52	0.54	0.24-0.74	27	15 of 27
gait score	1	Xing2 18 mo	Xing2 6 mo	0.59	0.62	0.40-0.79	9	n.d.	0.64	0.69	0.46-0.78	6	n.d.
gait analysis	16	Seburn2 18 mo	Seburn2 6 mo	0.56	0.56	p: 0.49–0.61	3	n.d.	0.52	0.51	p: 0.43–0.62	3	n.d.
hematology	6	Peters4 24 mo	Peters4 6 mo	0.67	0.70	p: 0.56–0.78	11		0.64	0.64	p: 0.57–0.69	11	
WBC	1	Petkova1 24 mo	Petkova1 6 mo	0.49	0.52	0.29-0.91	11	6 of 11	0.55	0.57	0.36-0.72	11	7 of 11
immunology	10	Peters4 24 mo	Peters4 6 mo	0.60	0.60	p: 0.51–0.78	11		0.60	0.61	p: 0.47–0.79	11	
immunology	8	Petkova1 24 mo	Petkova1 6 mo	0.64	0.66	p: 0.55–0.78	11		0.64	0.66	p: 0.55–0.75	11	
grip strength	1	Seburn2 24 mo	Seburn2 6 mo	0.57	0.56	0.31-0.83	24	19 of 24 **	0.53	0.57	0.20-0.76	21	14 of 21

Data of the Mouse Phenome Database (https://phenome.jax.org) were used for the analysis in the case that the identical project examined the same mouse strains at two different ages (“6 months old” vs. “18-20 months old”; and “6 months old” vs. “24 months old”). Data sets with group sizes of n ≥ 5 of a given sex for a chosen strain in both age groups were used. The average numbers of mice per experimental unit are n = 7.2–8.7 for female early adults, n = 5.3–7.5 for female late adults, n = 6.5–8.6 for male early adults, and n = 6.3–8.1 for male late adults. The trait “gait analysis” with only three strains examined was included in the table due to the high number of parameters (n = 16) analyzed for this trait.

For each test parameter, the CV of the early adult group and the CV of the late adult group, and subsequently the ratio r = CV_late adult_/ (CV_late adult_ + CV_early adult_) was calculated separately for each mouse strain. Subsequently, the mean and the median of the r’s of all mouse strains of the parameter were calculated. Finally, for traits with more than one test parameter, the mean of the parameter means (see “mean”) and the mean of the parameter medians (see “median”) were calculated for the given trait. For the traits “bone”, “clinical chemistry”, and “hematology” of the comparison “6 months old” vs. “18-20 months old”, the separate results of all 3, 11, and 6 test parameters, respectively, are consecutively listed.

“p:” in the columns “range (strains)”: For the traits including more than one test parameter, the range of the r values of the parameters is given.

r > 0.5 (n): The number of strains where the ratio r = CV_late adult_/ (CV_late adult_ + CV_early adult_) is > 0.5. In the case that the late adults show the identical phenotypic variability as the early adults for a given parameter, 50% of the strains are expected to deliver r values > 0.5 (and the other 50% of the strains are expected to deliver r values < 0.5). The detected counts of r values > 0.5 and < 0.5 were statistically analyzed by using the chi-squared test (*, p < 0.05; **, p < 0.01; ***, p < 0.001).

f, female; m, male; CV, coefficient of variation = standard deviation/ mean; CHr, reticulocyte corpuscular hemoglobin; DXA, dual-energy X-ray absorptiometry; WBC, white blood cell count; n.d., not determined (as less than 10 strains were included in the analysis).

The RIKEN BioResource Research Center (RBRC) (https://ja.brc.riken.jp/lab/jmc/mouse_clinic/Aged_JMC/group_Condition-Aged_mice/index.html) additionally submitted online a comprehensive phenotypic screen of a small number of C57BL/6NJcl mice (n = 7–14 per sex) at an early (9–16 weeks) and late (49–56 weeks) age period.

The phenotypic variability of test parameters and traits was measured by calculating the coefficient of variation (CV = standard deviation/ mean) of animals with the same sex of a specific mouse strain. The CV of the early adult mouse group and the CV of the late adult mouse group were compared subsequently by the calculation of the ratio r = CV_late adult_/ (CV_late adult_ + CV_early adult_) for a given parameter/ trait. Data analysis was carried out using the software program Microsoft Excel 2016 (Microsoft Corp., Redmond, WA). The chi-squared test was used for the statistical analysis of the data.

## Results

In this study, the phenotypic variability of test parameters and traits was measured by calculating the coefficient of variation (CV = standard deviation/ mean) of animals with the same sex of a specific mouse strain. The CV of the early adult mouse group and the CV of the late adult mouse group were compared subsequently by the calculation of the ratio r = CV_late adult_/ (CV_late adult_ + CV_early adult_) for a given parameter/ trait. r = 0.5 if the late adults show the identical phenotypic variability as the early adults. Increase or decrease of the phenotypic variability of the late adults vs. the early adults leads to r values > 0.5 or < 0.5, respectively. In the case that the late adults show the identical phenotypic variability as the early adults for traits with more than one test parameter measured, 50% of the parameters of a given trait are expected to deliver r values > 0.5 (and the other 50% of the parameters are expected to deliver r values < 0.5).

For the following values of r = CV_late adult_/ (CV_late adult_ + CV_early adult_) the respective relative change of the phenotypic variability of the late adults vs. the early adults is given in parentheses: r = 0.25 (0.33 × phenotypic variability of the late adults compared to the early adults); 0.33 (0.5×); 0.4 (0.66×) 0.43 (0.75×); 0.47 (0.9×); 0.5 (1×); 0.52 (1.1×); 0.55 (1.25×); 0.6 (1.5×); 0.66 (2×); and 0.75 (3×). Thus, an increase of the CV of the late adults vs. the early adults of 50% or more results in r values of ≥ 0.6.

During the analysis, body weight as well as lean mass and fat mass as test parameters often turned out to a show a higher variability in the late age group vs. the young age group (see below). Therefore, further test parameters were compared between the age groups as absolute values without relation to body weight or body composition parameters. Data of four different databases were analyzed.

The JaxKOMP project (https://phenome.jax.org/centers/JaxKOMP) analyzed a high number of C57BL/6NJ mice with an age of 7–20 weeks (“early adult”) and 66–81 weeks (“late adult”; n between > 100 and > 500 per test parameter) with a great number of test parameters, and all results of the individual mice have been submitted online. Therefore, in the current study the 95% data range (by excluding 2.5% each of the highest and lowest values) was chosen for each parameter as physiological range to exclude technical outliers. For traits with more than one test parameter measured, the mean of the r’s of all test parameters of a given trait was calculated (Table 1).

Analogous results were found for most traits in both sexes. The tests resulted in following r values: body weight: > 0.6, with higher values for females; lean mass and fat mass: partly clearly over 0.5, with higher values for females; bone: 0.5 for females, and < 0.5 for males; clinical chemistry: nearly 0.5 (with 6 and 7 of the 13 test parameters showing values > 0.5 for females and males, respectively); glucose tolerance test: > 0.5; hematology: distinctly > 0.5 (with 6 of 7 test parameters showing values > 0.5 in both sexes); electrocardiogram: nearly 0.5; grip strength: < 0.5; behavioral tests (hole board, light-dark box, open field): 0.55 to 0.6 (see columns “mean” of Table 1). Between the traits, no relation was detected for the level of the CV value (see columns “cv (range)” of Table 1) and the r value (see columns “mean” of Table 1) of a given trait.

As there is no phenotypic parameter described as a control for the analysis of the variability in different mouse age groups, no substantial variability of different mouse age groups for the parameter “body length” was assumed as hypothesis, particularly as “body length” showed a low CV in examined mouse groups in published studies [3] as well as in this study (CV = 0.03 for all four mouse groups “female early adult”, “female late adult”, “male early adult”, and “male late adult”; see columns “cv (range)” of Table 1). The analysis of the JaxKOMP project data delivered r values of 0.52 and 0.49 for females and males, respectively (see columns “mean” of Table 1).

The trait “hematology” included seven parameters (see Materials and Methods section). The two parameters “WBC” and “platelets” showed much higher CVs within a given mouse group in both the JaxKOMP project and the IMPC LATE ADULT project (see below) which corresponds to published studies [3]. Therefore, subsequently separate r values were analyzed for the group of the five remaining hematology parameters usually showing a low CV (MCV, MHEM, MPV, RBC, RDW), as well as for the group “WBC, platelets”. “WBC, platelets” showed a higher r value, however, the r value of the group of the remaining five hematology parameters was still > 0.5 (see columns “mean” of Table 1).

The trait “clinical chemistry” included 13 test parameters and delivered a r value of nearly 0.5 both for females and males. This was confirmed by the fact that half of the parameters showed r values < 0.5 and > 0.5, respectively (see columns “r > 0.5 (n)” of Table 1). The separate analysis of the 13 parameters resulted in r values within a high range (see columns “range” of Table 1). As described in published studies [3] as well as in this study (see columns “cv (range)” of Table 1), the separate clinical chemistry parameters showed different CV values in examined mouse groups. Therefore, subsequently the clinical chemistry parameters were classified in the four subgroups “electrolytes” (calcium with low CV, phosphorus with higher CV), “plasma enzyme activities” (ALT, AST, and AP; with high CVs), “substrates low” (albumin and total protein; with lower CVs), and “substrates high” (bilirubin, cholesterol, creatinine, glucose, triglycerides, urea; with higher CVs). For both the JaxKOMP project and the IMPC LATE ADULT project (see below), no relation was detected for the level of the CV value and the r value of the four subgroups within the trait “clinical chemistry”. This was also true for the analysis of the 12 test parameters of the trait “electrocardiogram” after subsequently classifying them in two subgroups with low CVs and high CVs, respectively.

The behavioral tests (hole board, light-dark box, open field) resulted in r values between 0.55 and 0.6 (see columns “mean” of Table 1). All test parameters of the behavioral tests showed high to very high CVs (> 0.1 to > 1.0; see columns “cv (range)” of Table 1), therefore, no subsequent analyses with subgroups of parameters with lower CVs and higher CVs was carried out.

Different projects submitted data to the database IMPC LATE ADULT (https://www.mousephenotype.org/data/late-adult-data). Data of up to nine projects using wild-type animals of C57BL/6N substrains with an age of 8–16 weeks (“early adult”) and 49–63 weeks (“late adult”) were analyzed (Table 2). Data sets were included with at least 50 mice of a given sex examined as “late adult” group for a test parameter. The test parameters chosen from the database are listed in S2 Table. For traits with more than one test parameter measured, the mean and the median of the r’s of all test parameters within a given trait of a project was calculated. Subsequently, the mean was calculated from the project means (see columns “mean” of Table 2).

Following r values were found in traits with data from at least three projects: body weight, lean mass and fat mass: > 0.5 for females, and around 0.5 for males; bone: > 0.5; clinical chemistry: slightly over 0.5; intraperitoneal glucose tolerance test: > 0.5; hematology: > 0.5 for females and 0.5 for males; immunology (n = 2 projects): > 0.5; echocardiography and electrocardiogram: around 0.5; grip strength: < 0.5; fear conditioning (n = 2 projects): < 0.5; light-dark box (n = 1 project): < 0.5 for females and 0.5 for males; open field, rotarod (n = 1 project) and Y-maze (n = 1 project): > 0.5 (see columns “mean” of Table 2). Individual phenotypic trait data of all projects including n ≥ 100 mice per sex for the late adult group of the database IMPC LATE ADULT are presented in further detail in S1 Table.

4-5 and 2 projects analyzed the parameters “body length” and “tibia length”, respectively. As already stated above for the analysis of the data sets of the JaxKOMP project, “body length” was hypothetically chosen to serve as potential “control” test parameter. The CVs of both parameters “body length” and “tibia length” for all four mouse groups “female early adult”, “female late adult”, “male early adult”, and “male late adult” of all projects were ≤ 0.06 (see also columns “CV” of S1 Table). The mean r values were 0.48 and 0.44 for “body length”, and 0.49 and 0.51 for “tibia length” in females and males, respectively (see columns “mean” of Table 2). However, the r values of individual projects for “body length” remarkably deviated to a high extent from the hypothetized r value of 0.5 (see columns “range (projects)” of Table 2), also in projects including n ≥ 100 mice per sex for the late adult group (S1 Table). The review of the downloaded values of mean and standard deviation (SD), which are published online, gave hints for the assumption that a rounding error due to only one decimal digit for the SD value being specified can not be excluded in these cases. In total, the retrospective analysis does not support the choice of “body length” to serve as “control” test parameter for all projects where data sets were examined for this study.

As already described above for the JaxKOMP project, the subsequent analyses of subgroups for particular phenotypic traits was also carried out with the data of the IMPC LATE ADULT project. In the trait “hematology”, the separate analysis of “WBC, platelets” resulted in r values > 0.5, whereas the analysis of the group of the remaining parameters with lower CVs obtained r values around 0.5 for both sexes (see columns “mean” of Table 2). For the trait “clinical chemistry”, 12–20 test parameters were chosen from five different projects. Analogous to the results in the JaxKOMP project, the subsequent analyses of the four subgroups “electrolytes” (with low CVs, except of phosphorus and iron), “plasma enzyme activities” (with high CVs), “substrates low” (with lower CVs), and “substrates high” (with higher CVs) obtained no relation for the level of the CV value and the r value of the four subgroups of the trait “clinical chemistry” within a given project. This was also true for the analysis of the test parameters of the trait “electrocardiogram” after subsequently classifying them in two subgroups with low CVs and high CVs, respectively. For the five behavioral tests (fear conditioning, light-dark box, open field, rotarod, Y-maze), data from at least three different projects were collected only for the trait “open field” (n = 4 projects). Almost all test parameters of the behavioral tests showed high to very high CVs (> 0.1 to > 1.0; see columns “CV” of S1 Table), therefore, no subsequent analyses with subgroups of parameters with lower CVs and higher CVs was carried out.

Furthermore, data of the Mouse Phenome Database (MPD, https://phenome.jax.org) were used for the analysis in the case that the identical project examined the same mouse strains at two different ages (“6 months old” vs. “18-20 months old”; and “6 months old” vs. “24 months old”) (Table 3). Data sets with group sizes of n ≥ 5 of a given sex for a chosen strain in both age group were used. All data collected were derived from inbred strains. For each test parameter, the mean and the median of the r’s of all mouse strains were calculated. Subsequently, for phenotypic traits with more than one test parameter, the mean of the parameter means (see columns “mean” of Table 3) and the mean of the parameter medians (see columns “median” of Table 3) were calculated for the given trait. The number of the mouse strains involved greatly varied for the different traits (n = 6–27; see columns “n strains” of Table 3).

For the comparison “6 months old” vs. “18-20 months old” following r values were found: body weight, lean mass and fat mass: > 0.5, with higher values for females; bone: > 0.5 for females and 0.5 for males; clinical chemistry, hematology, and immunology: clearly > 0.5; electrocardiogram: slightly > 0.5; grip strength: slightly > 0.5; gait analysis: > 0.5. For the comparison “6 months old” vs. “24 months old”, the traits hematology and immunology showed an even higher increase of the variability when compared to the analogous results of the comparison “6 months old” vs. “18-20 months old” (see columns “mean” of Table 3).

All individual parameters – irrespective of their CV values – generally showed a very high range of the r values of the various mouse strains tested (see columns “range (strains)” of Table 3). It is assumed that this does not or only to a minor extent reflect the genetical differences of the mouse strains, but is predominantly caused by the low average numbers of mice per experimental unit (n = 5–9). Thus, only a high number of mouse strains tested for a given parameter may lead to representative mean r values in the MPD project. The singular results of each strain with the low number of tested mice clearly showed the appearance of unpredictable interactions between genotype, environment and experiment.

The “control” test parameter “body length” was analyzed in one project of the comparison “6 months old” vs. “20 months old”, and showed mean r values of 0.61 and 0.55 for females (23 strains examined) and males (19 strains examined), respectively (see columns “mean” of Table 3). The same investigator also carried out the comparison “6 months old” vs. “12 months old” for the parameter “body length”; use of these data resulted in mean r values of 0.51 and 0.54 for females (24 strains examined) and males (18 strains examined), respectively. The generally described very high range of the r value for the various mouse strains tested was also seen for “body length” (see columns “range (strains)” of Table 3).

Analysis of data of a relatively small number of C57BL/6NJcl mice with an age of 9–16 weeks (“early adult”) and 49–56 weeks (“late adult”) from the RIKEN BioResource Research Center (RBRC) (https://ja.brc.riken.jp/lab/jmc/mouse_clinic/Aged_JMC/group_Condition-Aged_mice/index.html) is shown in S3 Table. For each test parameter, the CV of the early adult group and the CV of the late adult group, and subsequently the ratio r = CV_late adult_/ (CV_late adult_ + CV_early adult_) was calculated. For traits with more than one test parameter measured, the mean and the median of the r’s of all parameters within a given trait was calculated (see columns “mean” of S3 Table).

Following r values were found: body weight, analyzed in various phenotypic analyses: mostly clearly > 0.5, with higher values for females; lean mass: > 0.5; fat mass: clearly > 0.5 for females, and clearly < 0.5 for males; clinical chemistry: around 0.5 for females and 0.53 for males; hematology: > 0.5; grip strength, open field: inconsistent results for both sexes (see columns “mean” of S3 Table). As observed in the outcome of the analysis of the phenotypic variability from data of the Mouse Phenome Database (see above), in respect of the phenotypic variability caution should be taken in the interpretation of results derived from singular mouse strains with only low numbers of animals examined.

## Discussion

The retrospective analysis of the phenotypic variability of test parameters was carried out with data sets of early adult mouse groups versus late adult mouse groups from projects which determined the age ranges for early adult groups and late adult groups for their own. Thus, the chosen data sets have not been produced especially for the analysis of the age-related phenotypic variability which is an inherent limitation of the study. Data from four databases were analyzed, where C57BL/6N substrain mice were used except of project MPD. The projects JaxKOMP, IMPC LATE ADULT, and RBRC delivered data from early adults (7–20 weeks old) vs. late adults (around 1.5 years old (JaxKOMP), and around 1 year old (IMPC LATE ADULT, and RBRC)). MPD data were collected for the comparison of the age groups “6 months old” vs. “18-20 months old”, and “6 months old” vs. “24 months old”. For the old mice of the MPD project, it is assumed, that only mice with no apparent symptoms of the pre-mortal geriatric stage were chosen for the analyses. Attempts have been made to relate different mouse age periods to human age [4]. The projects JaxKOMP and IMPC LATE ADULT examined high numbers (n ≥ 50) of C57BL/6N substrain mice. On the other hand, the small group sizes used in the projects MPD and RBRC may represent the relatively low numbers of animals which are usually used as group size at least in fundamental biomedical research.

Taking into account particularly the projects with high numbers of mice examined, in summary the comparison of late adults vs. early adults resulted in following r values: body weight including lean mass and fat mass for females: consistently > 0.5; body weight including fat mass for males: around 0.5 or > 0.5; clinical chemistry: often around 0.5, or > 0.5; hematology and immunology: often > 0.5, with higher values for females; echocardiography and electrocardiogram: around 0.5; grip strength: < 0.5 or around 0.5; behavioral tests: often > 0.5. The results also showed the appearance of unpredictable interactions between genotype, environment and experiment regarding the variability of the parameters and traits in the different projects analyzed.

Unsatisfactory validity and reproducibility of results obtained from biomedical research in mice are of major and constant scientific concern (e.g., see [5–7], and refs. therein). Thus, despite of elaborate efforts to standardize main known abiotic and biotic experimental factors, non-reproducible and inconsistent results still occur in present studies. In addition, the data sets chosen for this age-related analysis were inherently derived from long-term projects which harbour an increased risk of change of abiotic and biotic factors during the project (e,g. personnel interacting with the experimental animals) compared to short-term experiments. Inconsistent results of the age-related phenotypic variability between the projects observed in this retrospective study also hinder or negatively impact the study of the causes underlying the results of the analysis about age-related phenotypic variation. Thus, preceding experiments of the impact of main abiotic and biotic factors on the outcome of the analysis of age-related phenotypic variation would be beneficial.

The four projects differed in the choice of the specific age of the early adult and late adult mice; in addition, the chosen age ranges of the early adult and late adult mice often covered a large time interval in the projects except of project MPD. The high variation of the age in the early adult groups (JaxKOMP: 7–20 weeks; IMPC LATE ADULT; 8–16 weeks; RBRC; 9–16 weeks) may negatively interfere with the aim of this study to compare the phenotypic variability in early adult mice and late adult mice as the first part of the chosen time range may still cover the phase of the late adolescence of the animals [1,2]. Thus, these early adult groups may include putative differences in the phenotypic variability of late adolescent mice and adult mice. However, even in cases of parameters with different mean values between age subgroups within a chosen age group, this does not indicate for differences of the phenotypic variability between these age subgroups.

The use and separate analysis of both sexes in biomedical research is strongly recommended because of possible differences in the outcome [8–10]. It is assumed that the data sets have been achieved by using the housing method which is usually carried out when working with mice in biomedical research, i.e., both sexes are group housed, and females in their reproductive lifespan are used without regard to the stage of the estrous cycle. A meta-analysis revealed that group housing of mice increased the variability in both males and females by 37% [11]. Therefore, no sex is expected to take advantage of this housing method in respect to the extent of the variability compared to the other sex. The similar increase of the variability in group housed males and females is also expected to cover the consequences of the Lee Boot effect which leads to the suppression of the estrous cycle in group housed female mice [12]. In addition, the behavior of female mice was described to reflect individual identity far more than estrous state [13]. Thus, the appearance of a higher age-related phenotypic variability of females for some parameters found in this study can not be explained by well-reproduced results of the published research. On the other hand, it is assumed, that single housing of mice may have been necessary in the late adult groups of the four projects more frequently for males than for females due to the appearance of social incompatibilities in the group. This would lead to inconsistencies of the impact of the housing method in early adult groups and late adult groups especially for males.

At several time points across the lifespan of *Mus musculus*, linear and nonlinear shifts in gene expression during ageing were revealed [14,15]. In a retrospective analysis of a high number of humans, individual patient-specific set points of hematological parameters were identified which persisted for at least 20 years [16]. On the other hand, the analysis of epigenetic, transcriptomic, and metabolomic levels in mice indicated the occurrence of rapid changes of the biological age in both directions at least in various models of severe stress [17].

The phenotypic variability within the experimental unit determines the choice of the group size which is necessary for achieving valid and reproducible results. Thus, the results may also be considered for the calculation of the group size when using older mice. Depending on the difference which is determined as relevant for the test group vs. the control group, an increase of the phenotypic variability CV (= standard deviation/ mean) of 50% (which results in a ratio r = CV_late adult_/ (CV_late adult_ + CV_early adult_) = 0.6 for late adult mice) within the experimental unit leads to the need of using roughly about twice the animal number of late adult mice for gaining the identical statistical strength when compared to early adult mice which are usually used in biomedical research [3].

## Conclusions

Overall, the comparison of the late adults vs. early adults resulted in a higher phenotypic variability almost consistently for body weight including lean mass and fat mass for females as well as for hematology and immunology parameters, particularly for females. Clinical chemistry often appeared inconspicuous. No noticeable differences were detected for the traits echocardiography and electrocardiogram, whereas late adults also often showed a higher phenotypic variability for behavioral tests. The results also clearly showed the appearance of unpredictable interactions between genotype, environment and experiment regarding the variability of the parameters and traits in the different projects analyzed.

## Supporting information

S1 Table Variability of test parameters from projects including n ≥ 100 mice per sex for the late adult group of the database IMPC LATE ADULT. (XLSX)

S2 Table Test parameters chosen from the nine projects (n ≥ 50 mice per sex of the old age group analyzed) of the database IMPC LATE ADULT shown in Table 2. (XLSX)

S3 Table Variability of test parameters from C57BL/6NJcl mice (n = 7–14 per sex) of two different age groups of the RBRC project. (XLSX)
